# MicroRNAs in porcine uterus and serum are affected by zearalenone and represent a new target for mycotoxin biomarker discovery

**DOI:** 10.1038/s41598-019-45784-x

**Published:** 2019-06-28

**Authors:** Bertrand Grenier, Matthias Hackl, Susanna Skalicky, Michaela Thamhesl, Wulf-Dieter Moll, Roger Berrios, Gerd Schatzmayr, Veronika Nagl

**Affiliations:** 1 BIOMIN Research Center, Technopark 1, 3430 Tulln, Austria; 2 TAmiRNA GmbH, Muthgasse 18, 1190 Vienna, Austria; 3 BIOMIN Holding GmbH, Erber Campus 1, 3131 Getzersdorf, Austria

**Keywords:** miRNAs, Endocrine reproductive disorders, Diagnostic markers

## Abstract

The mycotoxin zearalenone (ZEN) poses a risk to animal health because of its estrogenic effects. Diagnosis of ZEN-induced disorders remains challenging due to the lack of appropriate biomarkers. In this regard, circulating microRNAs (small non-coding RNAs) have remarkable potential, as they can serve as indicators for pathological processes in tissue. Thus, we combined untargeted and targeted transcriptomics approaches to investigate the effects of ZEN on the microRNA expression in porcine uterus, jejunum and serum, respectively. To this end, twenty-four piglets received uncontaminated feed (Control) or feed containing 0.17 mg/kg ZEN (ZEN low), 1.46 mg/kg ZEN (ZEN medium) and 4.58 mg/kg ZEN (ZEN high). After 28 days, the microRNA expression in the jejunum remained unaffected, while significant changes in the uterine microRNA profile were observed. Importantly, 14 microRNAs were commonly and dose-dependently affected in both the ZEN medium and ZEN high group, including microRNAs from the miR-503 cluster (i.e. ssc-miR-424-5p, ssc-miR-450a, ssc-miR-450b-5p, ssc-miR-450c-5p, ssc-miR-503 and ssc-miR-542-3p). Predicted target genes for those microRNAs are associated with regulation of gene expression and signal transduction (e.g. cell cycle). Although the effects in serum were less pronounced, receiver operating characteristic analysis revealed that several microRNA ratios were able to discriminate properly between non-exposed and ZEN-exposed pigs (e.g. ssc-miR-135a-5p/ssc-miR-432-5p, ssc-miR-542-3p/ssc-miR-493-3p). This work sheds new light on the molecular mechanisms of ZEN, and fosters biomarker discovery.

## Introduction

During the last decades, the potential health risks deriving from endocrine active substances in food and feed have moved into the spotlight of public, scientific and regulatory attention^1^. This heterogeneous group may comprise hundreds of environmental chemicals, such as bisphenol A, phthalates, atrazine, polychlorinated biphenyls, polybrominated diphenyl ethers or dichlorodiphenyltrichloroethane. Typically, endocrine disruptors interfere with the endocrine system either by binding to hormone receptors (as agonist or antagonist), modulation of hormone receptor expression or affecting levels of circulating hormones^2^. Synthetic or natural compounds mimicking or blocking endogenous estrogens are referred to as xenoestrogens^3^.

The mycotoxin zearalenone (ZEN), a frequent contaminant of cereal-based food and feed, acts as full and partial agonist on estrogen receptors α and β (ERα, ERβ)^4^. Yet, the impact of ZEN on the endocrine system is not limited to ERα and ERβ activation but affects different targets in the hypothalamic-pituitary-gonadal axis^5^. For example, ZEN was described to modulate pituitary gonadotropin synthesis and secretion via G protein-coupled estrogen receptor 1 (GPER1)^6,7^. ZEN is also a suspected trigger factor for central precocious puberty in prepubertal girls^8^. As summarized by Fink-Gremmels and Malekinejad^4^, ZEN might additionally influence the synthesis of steroid hormones (e.g. by substrate competition for hydroxysteroid-dehydrogenases), impair the metabolism of endo- and xenobiotics (via activation of the pregnane X receptor), and cause developmental disorders in the offspring.

Among animals, pigs are considered to be the species most sensitive to ZEN, which is attributed to both the high proportion of cereals in their diet and species-specific differences in phase I metabolism^4,9^. In a recent survey, 88% of feed samples were contaminated with ZEN, reaching maximum levels of more than 11,000 µg/kg^10^. Piglets and prepubertal gilts are especially prone to ZEN-induced effects^9^, which is reflected in the recommendations of the European Commission for maximum ZEN levels in feed^11^. Clinical signs of ZEN in pigs include swelling and reddening of the vulva, enlargement of the uterus, ovarian atrophy, decreased fertility and stillbirth^4^. Despite ongoing research, certain molecular mechanisms of ZEN remain unclear, and the diagnosis of suspected cases of ZEN-induced disorders in pigs is challenging due to the absence of appropriate biomarkers^12,13^.

In this respect, studying the microRNA response to ZEN exposure might provide important insights in cellular mode of action of this mycotoxin. MicroRNAs constitute a specific class of small (approximately 22 nucleotides long) non-coding RNAs. They regulate gene expression at post-transcriptional level by mRNA cleavage or translational repression^14^. Due to the release of microRNAs from tissue to extracellular biofluids, microRNAs have remarkable potential for biomarker development. Measurement of circulating microRNAs, e.g. in serum, urine or saliva, can serve as indicator for pathological processes in tissues. Thus, microRNAs represent a new generation of predictive biomarkers for toxin exposure and disease^15,16^. The augmented interest in microRNAs as biomarkers is for instance illustrated by the constantly rising numbers of publications: while a PubMed search on “microRNA AND biomarker” resulted in 52 publications in 2007, it yielded to 2758 hits in 2017.

Altered microRNA levels have been observed in estrogen-related diseases, such as breast cancer or endometrial cancer^16,17^. The impact of estradiol (E_2_), known as an agonist of the ERs, on microRNA expression has been extensively studied in breast cancer cell lines, revealing a number of up- and down-regulated microRNAs upon E_2_ exposure (summarized by^17,18^). At the same time, microRNAs can lead to dysregulation of estrogen-related gene expression. In contrast, research on the impact of endocrine disruptors on microRNA expression is still in its infancy^17,19^. For ZEN, only two reports are available, both focusing on pigs and employing targeted approaches for microRNA quantification (qPCR on a few selected microRNAs)^20,21^. Recently, the increased usage of ‘omics´-technologies was proposed for addressing existing mechanistic knowledge gaps in endocrine disruptor research^22^. By using high-throughput platforms, RNA sequencing (RNA-Seq) allows the identification and quantification of the complete set of transcripts of a cell/tissue^23^. As a total of 451 microRNAs are currently annotated in swine (miRBase 22, www.mirbase.org)^24^, small RNA-Seq is the method of choice to generate a comprehensive view on alterations of microRNAs.

The aim of our study was to examine the impact of dietary ZEN exposure on the global microRNA expression in uterus and jejunum. Subsequently, selected microRNAs were measured in serum, and used for receiver operating characteristic (ROC) curve analysis and target gene prediction, respectively. By employing untargeted and targeted transcriptomics approaches, our work contributes substantially to the elucidation of the mode of action of ZEN.

## Results

### Clinical signs

**Figure 1:**
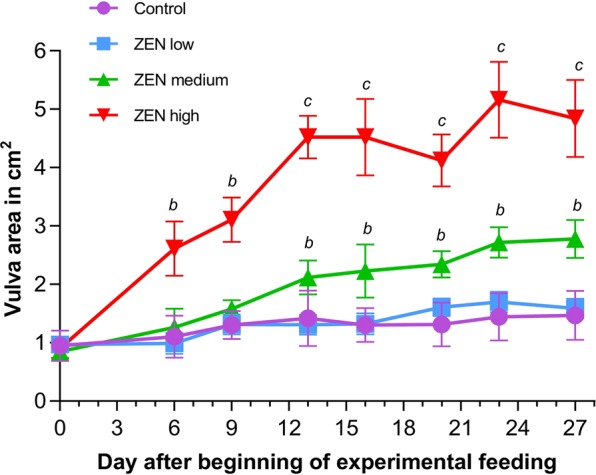
Effect of increasing dietary ZEN concentrations on vulva area of piglets. Vulva area (mean ± standard deviation, n = 6) of piglets exposed to uncontaminated feed (Control) or feed containing 0.17 mg/kg ZEN (ZEN low), 1.46 mg/kg ZEN (ZEN medium) and 4.58 mg/kg ZEN (ZEN high) was calculated after measuring the vulva length (in cm) and width (in cm) in regular intervals. Significant differences between treatment groups and for each time point are indicated by letters^b,c^ with^a^ for Control and ZEN low groups for every time point but not displayed (Tukey’s multiple comparison following two-way repeated measures ANOVA, p < 0.05).

To monitor the effects of increasing dietary ZEN concentrations (0.17 mg/kg, 1.46 mg/kg, 4.58 mg/kg) on the reproductive tract, the vulva area of individual piglets was measured in regular intervals during the experimental period. As illustrated in Fig. 1, ZEN exposure caused a dose-dependent increase of the vulva area, which was significant compared to the Control group from day 13 (ZEN medium) or day 6 (ZEN high) onwards. On day 27, the vulva area was enlarged by a factor of 1.9 and 3.3 in the ZEN medium and ZEN high group, respectively. In contrast, no significant differences were observed between the ZEN low and Control group until the end of the experiment.

Likewise, the impact of ZEN exposure on the weight of the reproductive tract was more pronounced at the end of the experiment. The average reproductive tract weight, expressed in (g per kg body weight)*100, was 51.8 ± 20.6 for the Control group, 55.8 ± 17.2 for the ZEN low group, 121.4 ± 43.4 for the ZEN medium group, and 353.4 ± 110.6 for the ZEN high group. Independent of the concentration, ZEN exposure had no effect on the body weight of the piglets (data not shown).

### Small RNA sequencing in tissues

To generate microRNA expression profiles in 18 uterus and 18 jejunum samples, RNAs with sizes between 18 and 50 nucleotides were selected for sequencing. The total number of reads exceeded 10 Mio in 34 out of 36 samples (≈5Mio reads reached in two samples). A peak for the read counts was observed at ≈21/22 nucleotides, which corresponds to the presence of mature microRNAs in the data. The reads were mapped to the miRBase database, and 40 to 75% of the reads deriving from uterus samples were identified as microRNAs, which is within the regular observations made for tissue RNA (Supplementary Figs S1 and S2). The same pattern was observed for jejunum tissue, with the exception of one sample from the ZEN high group, for which <25% of the reads were assigned as microRNA. Reads that could not be assigned to known microRNAs in swine were subsequently mapped against mature miRNAs from other mammalian species to identify novel conserved miRNAs. This led to the identification of 11 additional miRNAs in uterus, and 10 in jejunum, respectively. Remaining reads were used for prediction of putative novel miRNAs using miRPara software^25^. On average, 97 thousand reads (0.5%) were annotated as putative novel microRNAs per sample. The number of unmapped reads was low for both tissues (<10% for all samples).

**Figure 2:**
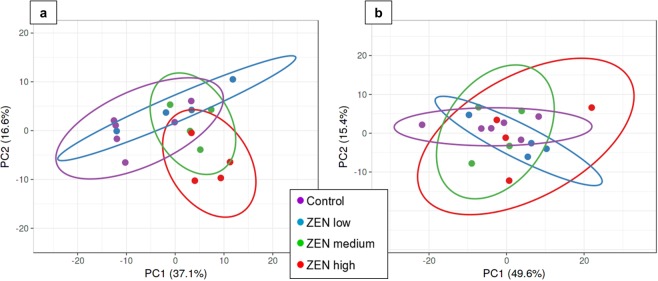
Principal component analysis on microRNA expression (>10 TPM) in uterus (**a**) and jejunum (**b**) of piglets exposed to uncontaminated feed (Control, n = 6)) or feed containing 0.17 mg/kg ZEN (ZEN low, n = 4), 1.5 mg/kg ZEN (ZEN medium, n = 4) or 4.6 mg/kg ZEN (ZEN high, n = 4).

To correct for sequencing depth, the TPM (Tags Per Million) normalization procedure was carried out. In total, 190 and 181 microRNAs with an expression level exceeding ten TPM were identified in uterus and jejunum, respectively. Principal component analysis (PCA) was performed on those microRNAs, revealing dietary treatment as major determinant for microRNA expression in uterus (Fig. 2a). While the Control and ZEN low groups shared a similar microRNA expression, the ZEN medium and ZEN high groups exhibited a markedly distinct pattern. In contrast, no clear clustering of the experimental groups was observed for the microRNA expression in jejunum (Fig. 2b).

Following PCA analysis, the differential expression analysis was done with EdgeR to identify microRNAs significantly affected upon ZEN exposure (with FDR adjusted p-value < 0.05). By doing so, no differentially regulated microRNAs were found comparing uterus samples of the ZEN low and Control groups. By contrast, 16 and 67 microRNAs were significantly affected in the ZEN medium and ZEN high groups, respectively (Table 1). In both groups, the majority of microRNAs was up-regulated: 13 out of 16 microRNAs in the ZEN medium group, and 41 out of 67 microRNAs in the ZEN high group were increased compared to the Control group. The most prominent changes in the expression of individual microRNAs were observed in the ZEN high group, especially on the microRNAs from the miR-503 cluster^26^ (miR-424-5p, miR-450a, miR-450b-5p, miR-450c-5p, miR-503, and miR-542-3p) with 6.0 to 12.6 fold increase.

**Table 1:** MicroRNAs significantly affected in the uterus of ZEN exposed piglets.

microRNA ID	log_2_(fold change) compared to Control			FDR adjusted p-value compared to Control			Coefficient r dose response^1^
	ZEN low	ZEN medium	ZEN high	ZEN low	ZEN medium	ZEN high	
**ssc-miR-542-3p**	**0**.**33**	**1**.**95**	**3**.**66**	**1**.**00**	**0**.**00**	**0**.**0000**	**0**.**98**
**ssc-miR-424-5p**	**0**.**30**	**1**.**68**	**3**.**34**	**1**.**00**	**0**.**01**	**0**.**0000**	**0**.**98**
ssc-miR-450c-3p	#N/A	#N/A	3.18	#N/A	#N/A	0.0000	#N/A
**ssc-miR-450c-5p**	**0**.**62**	**1**.**84**	**3**.**11**	**1**.**00**	**0**.**00**	**0**.**0000**	**0**.**98**
**ssc-miR-450b-5p**	**0**.**54**	**1**.**55**	**2**.**91**	**1**.**00**	**0**.**01**	**0**.**0000**	**0**.**99**
ssc-miR-424-3p	#N/A	#N/A	2.90	#N/A	#N/A	0.0000	#N/A
**ssc-miR-450a**	**0**.**40**	**1**.**56**	**2**.**87**	**1**.**00**	**0**.**00**	**0**.**0000**	**0**.**98**
**ssc-miR-503**	**0**.**17**	**1**.**22**	**2**.**59**	**1**.**00**	**0**.**03**	**0**.**0000**	**0**.**99**
ssc-miR-22-3p	0.61	0.65	1.69	1.00	0.13	0.0000	0.97
ssc-miR-758	−0.11	0.58	1.67	1.00	0.55	0.0001	0.99
**ssc-miR-143-5p**	**0**.**31**	**1**.**06**	**1**.**63**	**1**.**00**	**0**.**03**	**0**.**0000**	**0**.**95**
ssc-miR-493-3p	#N/A	0.81	1.63	#N/A	0.24	0.0001	#N/A
**ssc-miR-206**	**1**.**22**	**1**.**34**	**1**.**56**	**0**.**16**	**0**.**01**	**0**.**0041**	**1**.**00**
ssc-miR-493-5p	0.02	0.87	1.55	1.00	0.34	0.0002	0.96
ssc-miR-432-5p	−0.12	0.31	1.53	1.00	0.83	0.0001	1.00
ssc-miR-369	0.23	0.33	1.50	1.00	0.83	0.0000	0.98
ssc-miR-127	0.09	0.59	1.48	1.00	0.54	0.0001	1.00
ssc-miR-370	−0.09	0.90	1.46	1.00	0.23	0.0015	0.92
ssc-miR-136	0.04	0.18	1.45	1.00	0.93	0.0001	0.98
ssc-miR-140-3p	0.22	0.79	1.45	1.00	0.13	0.0000	0.98
ssc-miR-22-5p	0.25	0.63	1.44	1.00	0.21	0.0001	1.00
ssc-miR-146a-5p	0.21	0.05	1.36	1.00	0.97	0.0079	0.92
ssc-miR-182	0.18	0.80	1.33	1.00	0.21	0.0208	0.96
ssc-miR-183	0.30	1.05	1.29	1.00	0.08	0.0313	0.87
ssc-miR-378	0.23	0.83	1.29	1.00	0.08	0.0024	0.95
ssc-miR-455-3p	0.26	0.80	1.24	1.00	0.10	0.0011	0.96
ssc-miR-143-3p	0.18	0.74	1.21	1.00	0.12	0.0006	0.96
**ssc-miR-7135-3p**	**#N/A**	**0**.**99**	**1**.**19**	**#N/A**	**0**.**03**	**0**.**0045**	**#N/A**
ssc-miR-34a	0.07	0.33	1.17	1.00	0.83	0.0010	1.00
ssc-miR-455-5p	0.09	0.69	1.15	1.00	0.12	0.0005	0.95
**ssc-miR-497**	**0**.**32**	**1**.**06**	**1**.**14**	**1**.**00**	**0**.**02**	**0**.**0008**	**0**.**78**
ssc-miR-195	0.39	0.77	1.13	1.00	0.18	0.0016	0.97
**ssc-miR-1**	**0**.**88**	**1**.**04**	**1**.**12**	**0**.**20**	**0**.**03**	**0**.**0042**	**0**.**92**
ssc-miR-133a-5p	0.64	1.01	1.12	1.00	0.09	0.0138	0.86
ssc-miR-145-5p	0.09	0.60	1.06	1.00	0.37	0.0013	0.96
ssc-miR-142-3p	−0.42	−0.15	1.02	1.00	0.90	0.0452	0.99
ssc-miR-144	0.12	0.27	0.98	1.00	0.84	0.0251	0.99
ssc-miR-451	0.12	0.05	0.92	1.00	0.97	0.0182	0.93
ssc-miR-21	−0.04	0.30	0.86	1.00	0.78	0.0208	1.00
ssc-miR-323	0.17	0.39	0.83	1.00	0.83	0.0294	1.00
ssc-miR-486	0.46	0.36	0.82	1.00	0.76	0.0339	0.88
ssc-miR-490-3p	0.28	1.06	0.50	1.00	0.03	0.2623	0.04
ssc-miR-490	−0.10	1.11	0.37	1.00	0.03	0.4416	0.15
ssc-miR-221-3p	0.02	−0.21	−0.72	1.00	0.83	0.0342	−1.00
ssc-miR-664-3p	−0.04	−0.46	−0.74	1.00	0.47	0.0421	−0.94
ssc-miR-874	−0.21	−0.12	−0.78	1.00	0.95	0.0251	−0.92
ssc-miR-218b	0.09	−0.22	−0.79	1.00	0.83	0.0314	−1.00
ssc-miR-24-2-5p	−0.13	−0.19	−0.80	1.00	0.84	0.0251	−0.98
ssc-miR-218	0.12	−0.26	−0.80	1.00	0.83	0.0318	−0.99
ssc-miR-218-5p	0.14	−0.28	−0.82	1.00	0.83	0.0265	−0.99
ssc-miR-26a	0.03	−0.40	−0.83	1.00	0.73	0.0379	−0.97
ssc-miR-92a	−0.10	−0.27	−0.89	1.00	0.83	0.0076	−1.00
ssc-miR-30e-3p	−0.04	−0.26	−0.89	1.00	0.83	0.0083	−1.00
ssc-miR-1468	−0.17	−0.26	−0.94	1.00	0.84	0.0143	−0.98
**ssc-miR-335**	**0**.**19**	**−1**.**17**	**−0**.**94**	**1**.**00**	**0**.**03**	**0**.**0385**	**−0**.**61**
ssc-miR-99a	−0.20	−0.06	−0.97	1.00	0.97	0.0042	−0.91
ssc-miR-100	−0.09	−0.34	−0.98	1.00	0.73	0.0024	−1.00
ssc-miR-10b	0.15	−0.62	−1.02	1.00	0.31	0.0091	−0.91
ssc-miR-125b	−0.08	−0.30	−1.02	1.00	0.78	0.0010	−1.00
ssc-miR-146b	0.06	−0.42	−1.04	1.00	0.66	0.0059	−0.99
ssc-miR-708-3p	−0.52	−0.16	−1.09	1.00	0.93	0.0203	−0.77
**ssc-miR-181c**	**−0**.**05**	**−0**.**95**	**−1**.**17**	**1**.**00**	**0**.**03**	**0**.**0029**	**−0**.**84**
ssc-miR-125a	−0.07	−0.40	−1.28	1.00	0.73	0.0001	−1.00
ssc-miR-129b	0.09	0.18	−1.37	1.00	0.90	0.0006	−0.94
ssc-miR-149	−0.06	−0.44	−1.50	1.00	0.66	0.0001	−1.00
ssc-miR-215	−0.96	−0.12	−1.60	1.00	0.97	0.0251	−0.63
ssc-miR-129a	0.49	−0.54	−1.61	1.00	0.66	0.0022	−0.97
**ssc-miR-187**	**−0**.**52**	**−1**.**78**	**−1**.**90**	**1**.**00**	**0**.**01**	**0**.**0037**	**−0**.**78**
ssc-miR-204	0.04	−0.52	−2.04	1.00	0.43	0.0000	−1.00

MicroRNAs highlighted in bold were significantly affected in the ZEN medium and ZEN high groups (FDR adjusted p-value < 0.05). The log2(fold change) is indicated for each experimental group with n = 4 for ZEN exposed groups and with n = 6 for the Control group.

^1^Coefficient dose response was estimated following correlation analysis between fold changes and increasing concentrations of ZEN. If the value of r is close to +1, this indicates a strong positive correlation, and if r is close to −1, this indicates a strong negative correlation.

**Figure 3:**
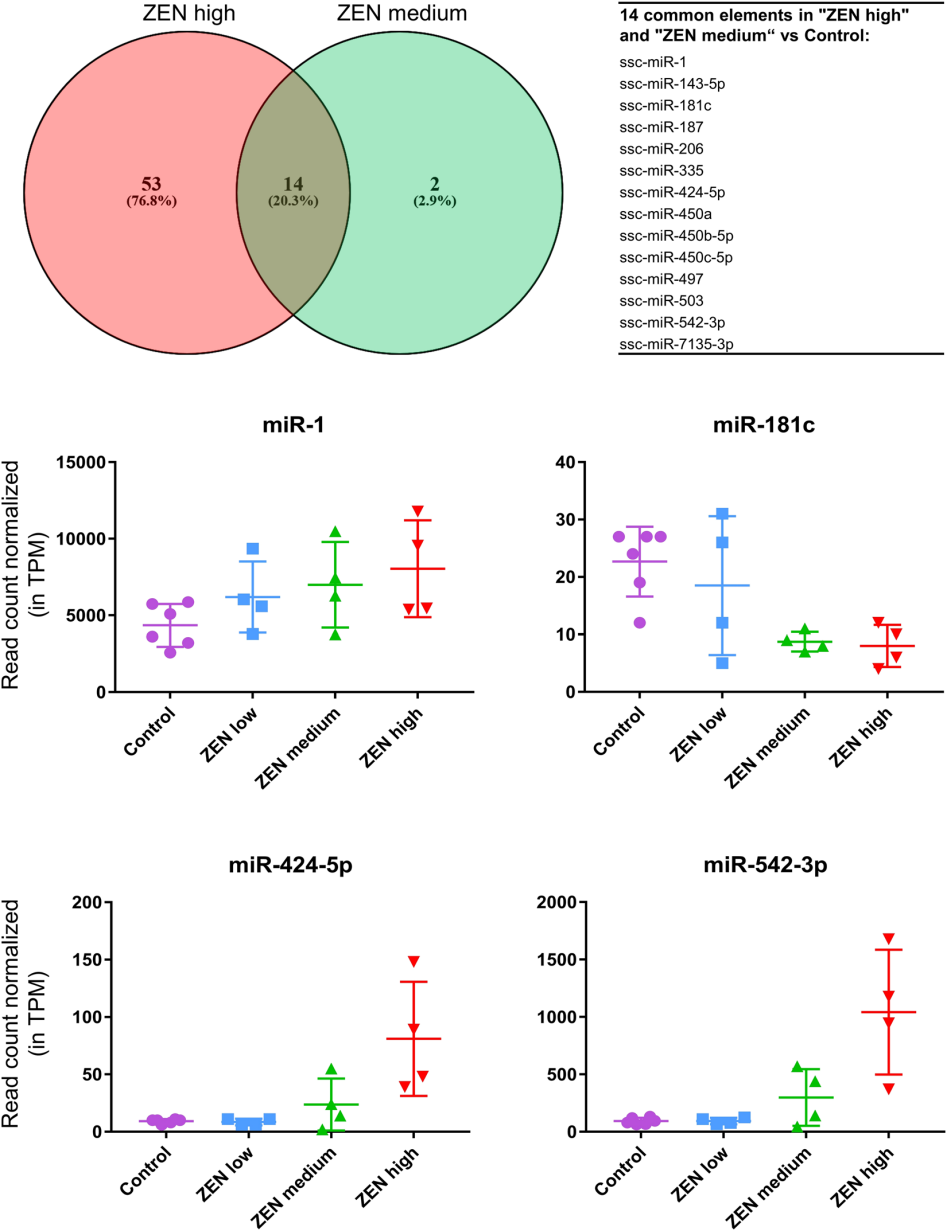
Effect of increasing dietary ZEN concentration on microRNA expression in the uterus. The Venn diagram (top) lists the 14 microRNAs commonly affected in the ZEN medium and ZEN high groups, and significantly different from the control group. As an example, the normalized read counts (tags per million, TPM) of miR-1, miR-181c, miR-424-5p, and miR-542-3p in the Control (n = 6) and ZEN exposed groups (n = 4 per group) are displayed.

As depicted in Fig. 3, fourteen microRNAs were commonly affected in the ZEN medium and ZEN high groups. While ssc-miR-1, ssc-miR-143-5p, ssc-miR-206, ssc-miR-424-5p, ssc-miR-450a, ssc-miR-450b-5p, ssc-miR-450c-5p, ssc-miR-497, ssc-miR-503, ssc-miR-542-3p and ssc-miR-7135-3p were up-regulated in both groups, ssc-miR-181c, ssc-miR-187 and ssc-miR-335 were down-regulated. In addition, ZEN exposure led to a significant increase or decrease of ten putative or predicted microRNAs in uterus (Supplementary Table S1). Among those, only one putative microRNA (put-miR-300) was significantly affected in both ZEN medium and ZEN high.

In line with results from the PCA analysis, no microRNAs were differentially regulated in jejunum samples from ZEN exposed piglets.

### Validation of small RNA-Seq results in uterus by qPCR

For confirmation of sequencing results, five microRNAs (ssc-miR-181c, ssc-miR-424-5p, ssc-miR-450c-5p, ssc-miR-503 and ssc-miR-542-3p) were quantified in the same uterus samples by qPCR. The correlation analysis (X,Y pairs) between fold changes observed in RNA-Seq and in qPCR showed a r Pearson coefficient of 0.97 when including the data from the five microRNAs and for each ZEN group (p < 0.0001; data not shown).

There was a poor correlation (r = 0.08 for 90 X,Y pairs; p = 0.48) between TPM in small RNA-Seq and Cq in qPCR for these five microRNAs. This is likely attributed to the ligation efficacy of the sequencing adaptors to the ends of certain microRNA sequence, with higher counts for microRNAs with “compatible” ends^27^. However, since ligation bias is the same for all experimental groups, the relative differences (=fold changes) are consistent.

### Target gene prediction

Since tools for *in silico* prediction of target genes had been mostly developed for model organisms (humans, rodents, and zebrafish) and the seed sequence may differ across species, the porcine sequences of our microRNAs of interest were first compared to the human homologs. The selected microRNAs (showing the most changes) had 100% homology on the seed sequence.

The prediction analysis was first divided in two separate analyses according to the initial effect seen on those microRNAs (up or down). For each microRNA, the list of target genes generated by the different sources of prediction software (miRTarBase, microT-CDS, TargetScan, and miRDB) was further processed by scoring each target gene based on prediction score and occurrence score. By doing so, the initially large number of microRNA-mRNA interaction sites was narrowed down to the most likely target genes (Table 2). The prediction analysis for the five up-regulated microRNAs (known to belong to the same cluster miR-503^26^) showed that 346 target genes were found at least in the prediction datasets of two of these microRNAs. Extra analysis on network prediction and interconnection of genes was done with miRNet and is included in Supplementary Fig. S3. Signaling pathway prediction tools revealed that these target genes were associated with regulation of gene expression and protein modifications, as well as signal transduction. This latter signaling pathway was also found enriched in the 65 target genes in common across the four down-regulated microRNAs (Table 2).

**Table 2:** Target gene prediction analysis on certain microRNAs found in common between ZEN medium and ZEN high groups, but significantly up- or down-regulated compared to control group.

Homolog microRNAs in humans for target gene prediction analysis^a^	Number of predicted target genes found:			
	With scoring method^b^	After filtering score > 17.0	In common in the prediction datasets of at least two microRNAs, and enrichment pathway analysis^c^	In common in the prediction datasets of at least three microRNAs, and gene name
**Up-regulated microRNAs** ^**d**^				
hsa-miR-542-3p	574	168	**346** • Regulation gene expression: **137/346** (e.g. RNA polymerase II transcription) • Regulation signal transduction: **101/346** (e.g. signaling in cancer, by BMP, by WNT) • Regulation of protein modification process: **110/346** (e.g. SUMOylation) • Immune system: **69/346** (e.g. signaling by interleukins)	**42** SPRED1, C1orf21, MOB4, CACUL1, PLAG1, SOCS6, CAPZA2, ASH1L, SALL1, UBN2, CBX4, CD2AP, SREK1, BZW1, SKI, TAOK1, RAB9B, NUFIP2, SLIT2, RAPH1, FBXW7, PTPN4, HELZ, COPS2, CCND2, AGO1, RTN4, ARMC8, OCRL, KIF5B, EPB41L1, ZNF367, HNRNPA1, CREBL2, TSC22D2, CRK, RAB3IP, ZBTB43, AKIRIN1, ZDHHC15, MAP3K7, GLP2R
hsa-miR-424-5p	1858	1054		
hsa-miR-503-5p	416	254		
hsa-miR-450a-5p	90	18		
hsa-miR-450b-5p	2180	1051		
**Down-regulated microRNAs** ^**d**^				
hsa-miR-181c-5p	1135	410	**65** • Disease of signal transduction: **17/65** (e.g. signaling by GPCR)	**5** THRB, SIRT1, MBNL1, PRLR, HIPK3
hsa-miR-187-3p	498	31		
hsa-miR-204-5p	786	261		
hsa-miR-135a-5p	939	481		

The up-regulated microRNAs reported in this table belong to the same cluster (miR-503 in humans).

^a^Sequences of mature microRNAs were compared between pigs and humans, and all of them have 100% homology on the seed sequence, and on the rest of the sequence as well (except 1 nucleotide change for miR-503-5p). ^b^Scoring method is based on prediction and occurrence scores for different databases (microT-CDS, TargetScan, miRDB, miRTarBase), and described in detail in the material and methods section. Further network prediction analysis can be found in Supplementary Fig. S3. ^c^Enrichment pathway analysis were performed with Gene Ontology Consortium, and Reactome Pathway Database. ^d^The target gene prediction analysis was carried out only with microRNAs showing at least fold changes >3 or < −3 between ZEN high and Control.

### Quantification of selected microRNAs in serum

As a follow-up, we evaluated whether circulating microRNAs reflect the tissue response observed after ZEN exposure. Besides the 14 microRNAs commonly affected in the ZEN medium and ZEN high groups, we selected 30 additional microRNAs based on sequencing results for quantification in serum by qPCR (Supplementary Table S2). One serum sample from the ZEN low group was excluded from the analysis because of hemolysis (n = 5; all other groups n = 6). For four of the tested microRNAs (ssc-miR-135, ssc-miR-187, ssc-miR-195, ssc-miR-497), the qPCR amplification was done with primers designed for the human homolog sequences (100% homology, Supplementary Table S2).

Several microRNAs had to be removed from the dataset due to insufficient signal amplification (ssc-miR-143-5p, ssc-miR-187, ssc-miR-493-5p, ssc-miR-708-3p, ssc-miR-7135-3p, ssc-miR-135b-5p, ssc-miR-301a-5p, put-miR-300). In general, most of the investigated microRNAs were expressed at low levels in serum (Cq > 33). Only eight microRNAs showed intermediate (Cq 30-33) or high (Cq < 30) expression (Supplementary Table S2). Certain microRNAs were not detectable in all treatment groups. For example, ssc-miR-133a-5p was found in only one out of six samples of the Control group, but in four out of five samples in the ZEN low, and in four out of six samples in the ZEN medium and ZEN high group, respectively.

**Figure 4:**
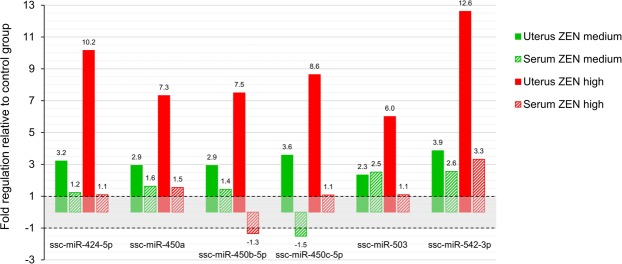
Comparison of the fold regulation (compared to Control) observed in the uterus and serum for the same microRNA for either ZEN medium or for ZEN high. The microRNAs displayed were the most up-regulated in the uterus of piglets fed ZEN, and are part of the same miR-503 cluster. Given the fold regulation cannot be between 1 and −1 (e.g. 0.5 fold change = 2 fold down-regulation), this zone is covered in grey.

First, the serum and tissue expression of the “core” 14 microRNAs (commonly affected in ZEN medium and ZEN high groups in uterus) was compared, revealing poor correlations for all tested microRNAs except for ssc-miR-542-3p (Pearson coefficient r = 0.42, p = 0.09 with log_2_NGS counts, delta Cq pairs). Only a few microRNAs (ssc-miR-1, ssc-miR-450a, ssc-miR-542-3p) exhibited a consistent up-regulation upon ZEN treatment in both uterus and serum. In contrast, ssc-miR-181c was up-regulated in uterus, but down-regulated in serum, while the opposite expression pattern was observed for ssc-miR-335. As an example, Fig. 4 displays the serum and tissue expression of the miR-503 cluster. Interestingly, when analyzing the 44 microRNAs, the effects of ZEN seemed to be more pronounced in the ZEN low and ZEN medium groups than in ZEN high group (e.g. ssc-miR-135a-5p, ssc-miR-181c, ssc-miR-432-5p, ssc-miR-493-3p, ssc-miR-503; Table 3). However, the overall effects of ZEN on circulating microRNAs were markedly less pronounced than in tissue.

**Table 3:** Fold regulation in the serum of 12 selected microRNAs (out of the 44 initially assessed).

microRNA ID	Fold regulation compared to Control		
	ZEN low	ZEN medium	ZEN high
ssc-miR-1	−3.74	2.78	1.35
ssc-miR-129a	−1.15	−2.32	−3.43
**ssc-miR-135a-5p**	2.04	**7**.**67**	2.70
**ssc-miR-181c**	1.74	**3**.**45**	1.78
ssc-miR-182	−1.43	1.05	−2.17
ssc-miR-206	−3.30	2.36	−2.65
**ssc-miR-432-5p**	**−6**.**97**	−3.50	−1.87
**ssc-miR-455-3p**	**−1**.**58**	1.43	1.42
**ssc-miR-455-5p**	−1.01	**2**.**62**	**1**.**85**
ssc-miR-493-3p	−2.41	−2.46	−1.98
ssc-miR-503	1.61	2.51	1.09
**ssc-miR-542-3p**	1.05	2.56	**3**.**32**

MicroRNAs highlighted in bold showed trend or significance in at least one of the ZEN groups compared to the Control (Fisher’s LSD test done on delta Cq with p < 0.1). The fold regulation is indicated for each experimental group with n = 6 for all groups, except n = 5 for ZEN low (excluded due to high hemolysis in one serum sample).

**Figure 5:**
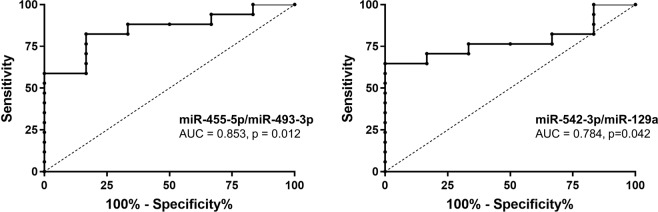
Receiver Operating Characteristic (ROC) curves and the area under the curve (AUC, with 95% CI) of the two most accurate predictor ratios ssc-miR-455-5p/ssc-miR-493-3p and ssc-miR-542-3p/ssc-miR-129a.

Finally, we performed ROC curve analysis to determine whether circulating microRNAs are suitable markers to differentiate between piglets non-exposed to ZEN (Control, n = 6) and piglets exposed to ZEN (ZEN low, ZEN medium and ZEN high, n = 17). To increase the sensitivity, the ROC analysis was not done on single microRNAs but on ratios between some up- and down-regulated microRNAs^28^. As demonstrated in Table 4 and Fig. 5, best results were obtained for ssc-miR-455-5p/ssc-miR-493-3p (AUC, Area Under the ROC Curve: 0.85, p < 0.01), with several other microRNA ratios showing AUC above 0.75.

**Table 4:** Receiver Operating Characteristic (ROC) analysis on selected microRNA ratios in the serum for discrimination between piglets non-exposed to ZEN (Control group) and piglets exposed to ZEN (ZEN low, ZEN medium, ZEN high).

microRNA ratios	AUC^*^	95% CI	*p-value*
ssc-miR-135a-5p/ssc-miR-1	0.706	0.419–0.993	0.146
ssc-miR-135a-5p/ssc-miR-129a	0.755	0.492–1.017	0.069
ssc-miR-135a-5p/ssc-miR-493-3p	0.755	0.536–0.974	0.069
ssc-miR-135a-5p/ssc-miR-206	0.696	0.441–0.951	0.161
ssc-miR-135a-5p/ssc-miR-432-5p	0.789	0.598–0.981	0.039
ssc-miR-455-5p/ssc-miR-1	0.657	0.362–0.951	0.263
ssc-miR-455-5p/ssc-miR-129a	0.755	0.539–0.971	0.069
ssc-miR-455-5p/ssc-miR-493-3p	0.853	0.692–1.014	0.012
ssc-miR-455-5p/ssc-miR-206	0.657	0.407–0.907	0.263
ssc-miR-455-5p/ssc-miR-432-5p	0.765	0.527–1.003	0.059
ssc-miR-542-3p/ssc-miR-1	0.598	0.349–0.847	0.484
ssc-miR-542-3p/ssc-miR-129a	0.784	0.598-0.970	0.042
ssc-miR-542-3p/ssc-miR-493-3p	0.774	0.576-0.974	0.049
ssc-miR-542-3p/ssc-miR-206	0.672	0.433-0.910	0.221
ssc-miR-542-3p/ssc-miR-432-5p	0.755	0.489-1.021	0.069

^*^AUC, Area Under the ROC Curve; CI, Confidence Interval.

## Discussion

MicroRNAs are considered as key regulators of many physiological processes and crucial player in the endocrine system^29^, and have gained increasing interest in biomarker research. Although studies on the presence and function of microRNAs in livestock are limited, they are discussed as markers for onset and progression of diseases in swine^30^. In addition, microRNAs are highly conserved across animal genomes, even between distantly related species, especially on the ‘seed’ region that is the main determinant of target specificity^16^. Here, we report for the first time the impact of a mycotoxin on microRNA expression in porcine jejunum and uterus, and the potential of circulating microRNAs as biomarker for ZEN-induced disorders.

Following ingestion, the gastrointestinal tract (GIT) is the first tissue exposed to ZEN. Compared to other mycotoxins, the effect of ZEN in the GIT seems to be limited^31,32^, with most effects seen in *in vitro* models. This observation on limited effects in the GIT could be related to the tissue distribution of estrogen receptors (ER) α and β. ZEN is known to be a full agonist of ERα, and ERα is highly expressed in uterus but less in the GIT (confirmed by qPCR in the current experiment; data not shown). ERβ appears to be the predominant ER type in the GIT, and the ratio of ERα vs β together with the multiplicity of receptors may determine the sensitivity to estrogens and its biological responses^33^. Recently, the effect of ZEN on the expression of seven microRNAs was investigated in the liver, duodenum, jejunum, ascending colon and descending colon of immature gilts^20^. Most pronounced alterations in microRNA expression were observed in the ascending colon after 42 days of exposure (miR-15a, miR-34a, miR-192), while effects in descending colon (miR-15a) and liver (miR-15a, miR-21, miR-192) were less evident. By contrast and in agreement with our findings, no significant changes on microRNA levels in jejunum were reported.

Conversely, ZEN elicited a marked dysregulation of uterine microRNA expression. In the ZEN high group, 67 microRNAs were affected, with fold-changes relative to the control group ranging from −4.1 to +12.6. As previously mentioned, literature on the effects of ZEN on microRNAs are scarce, and comprise only two studies focusing on selected microRNAs either in the porcine GIT or the pituitary gland. Still, in an attempt to compare our findings, we found two microRNAs described by Brzuzan *et al*.^20^ (miR-21 in liver, miR-34a in ascending colon) being significantly up-regulated in uterus samples of the ZEN high group. Besides, alterations in pituitary miR-7 expression have been reported after intraperitoneal ZEN administration (7.5 mg/kg b.w., equivalent to 150 mg/kg of feed) in ovariectomized piglets^21^. This particular microRNA was not affected in our trial, but the extremely high dose used, together with the different target tissue and route of administration do not allow appropriate comparison.

In our experiment, piglets fed the medium ZEN concentration of 1.46 mg/kg also showed significant changes of the uterine microRNA expression. Given dose-response is a hallmark for biomarker validation, we specifically focused on the 14 microRNAs commonly affected in the ZEN medium and ZEN high groups, and showing a dose response relationship (coefficient of dose response r ranging from 0.78 to 1.00 for up-regulated microRNAs, and from −0.61 to −0.84 for down-regulated microRNAs). For some of those, an involvement in the estrogen signaling has been demonstrated. For example, miR-503, which was significantly upregulated in our study following ZEN exposure, was recently addressed as “candidate master regulator of the estrogen response” following an integrative transcriptomics analysis (paired small RNA- and mRNA-Seq) in E_2_ (estradiol)-treated MCF-7 cells (human breast cancer cells)^34^. Deriving from the same cluster (a set of two or more microRNAs transcribed from physically adjacent microRNA genes) as miR-503, miR-424-5p was also significantly increased in the uterus samples of ZEN-exposed animals, and similarly reported to be up-regulated in MCF-7 and ZR-75.1 cells after E_2_ treatment^35^. MiR-424-5p has gained interest as human cancer marker, showing reduced expression levels in breast or cervix cancer, respectively^36,37^. Both microRNAs are discussed as potential anti-oncogenes, as they suppress cell proliferation by targeting either the ERα-regulatory circuit (via ZNF217^34^) or GPER^38^. In line with that, our target gene prediction analysis also identified ZNF217 as a major target for miR-503. This *in silico* prediction analysis was carried out with the five most up-regulated microRNAs (miR-424-5p, miR-450a, miR-450b-5p, miR-450c-5p, miR-503, and miR-542-3p) in the uterus of piglets fed ZEN, which have been recently reported to all belong to the aforementioned miR-503 cluster in humans, and linked to endometrial adenocarcinoma^26,39^. Due to large output in microRNA prediction analysis, the resulting list of target genes was further filtered excluding genes with low prediction scores and uncommon genes across microRNAs. The subsequent enrichment pathway analysis revealed a central role in signal transduction, especially in cancer. Interestingly, some enriched signaling pathways, such as Wnt and TGF-β1/Smad3, were also recently shown to be affected in the uterus and ovaries of piglets exposed to 1.5 mg ZEN/kg^40,41^. The signaling by BMP (top 1 signaling in our prediction analysis) includes different proteins from the Smad family that are the main signal transducers for receptors of the TGF-β superfamily, which are critically important for regulating cell proliferation. One could assume that the strong up-regulation of microRNAs from the miR-503 cluster would repress genes promoting cell growth in order to limit the enlargement of the reproductive tract caused by ZEN. A predicted target gene of this cluster (and other microRNAs in the present study) is Bcl-2, well known for its role in regulating cell death and classified as an oncogene. Bcl-2 is activated in response to E_2_, and this so-called genomic response to E_2_ is the result of the direct interaction between E_2_-liganded ER with the estrogen response element (15bp-palindrome ERE = 5′-AGGTCAnnnTGACCT-3′), a specific DNA sequence within the promoter of the Bcl-2 gene^42^. Interestingly, we found a similar palindromic motif in the promoter of Bcl-2 in pig (5′-TGGTCCnnnTGACCC-3′). We could hypothesize that ZEN turns on the transcription of this gene (and most likely others under ERE control), and as a response, the expression of microRNAs from the miR-503 cluster would be increased in order to target and repress Bcl2 expression^43^. Presence of ERE within microRNA genes, and their direct activation, is not to be excluded either.

Overall, ZEN resulted in the stimulation of uterine microRNA expression, with only three microRNAs being commonly down-regulated in both ZEN medium and ZEN high groups. Generally, down-regulation of microRNA expression is a hallmark of cancer as a probable consequence of mutations reducing the efficiency of the microRNA processing machinery^44^. However, unlike other mycotoxins, ZEN has not been reported mutagenic or carcinogenic, and it is also very unlikely that mutations occurred within such short time of exposure. Interestingly among the down-regulated microRNAs in the present study, ssc-miR-181c has been suggested together with members of the miR-181 family to play a role in embryo implantation and placentation in pigs, with ERα listed as one of the predicted target genes^45^. Moreover, the differential microRNA expression in ovaries between pigs with high or low litter size indicated that miR-181c possibly affects litter size^46^. *In vitro*, miR-181c was downregulated in MCF-7 cells treated with E_2_ or bisphenol A^47^, which is in line with our observations. Yet, comparisons between our findings and available *in vitro* results on (xen)estrogens are clearly hampered, not only by the extent that ZEN mimics the estradiol response and by the primarily usage of cancer cell lines, but also by conflicting results on the microRNA expression after E_2_ treatment^17^. Those differences might stem from variations in the employed methodology (e.g. for quantification of microRNAs), as well as from time-dependent dynamics in the microRNA response^34^ or the receptor status of cells. For example, Adams *et al*.^48^ found that both E_2_ and the ERα agonist propyl pyrazole triol markedly decreased the levels of miR-206 in MCF-7 cells. As part of a regulatory feedback mechanism, miR-206 can directly target ERα^48^ and ERα coactivator proteins^49^, thus suppressing estrogen response in MCF-7 cells. In contrast, treatment with selective ERβ-agonists led to increased miR-206 levels in MCF-7 cells^48^ and motoneurons^50^. Surprisingly, ZEN also caused a significant upregulation of uterine miR-206 level in our experiment. In principle, ZEN shows similar affinity to ERα and ERβ, but estrogenic potency is markedly reduced for ERβ, for which even anti-estrogenic activity was observed^51^. Previous report on a ZEN-induced shift of the ER ratio in uterus favoring ERβ expression^52^ could not be confirmed in the present study (data not shown). Hence, the observed increase of miR-206 cannot be explained by dominance of ERβ in this tissue. The interaction of ZEN and miR-206 warrants further investigation, especially due to the central role of this microRNA in estrogen signaling.

Interestingly, some microRNAs moderately increased following ZEN ingestion (regardless of the dosage used) were identified as part of the myomiR network (among them miR-206, but also miR-1, miR-133a, and miR-486^53^). The microRNAs from the myomiR family are known to be expressed specifically in the muscle, and to be involved in muscle cell differentiation. This is of interest considering the myometrium consists of smooth muscle. For other microRNAs altered upon ZEN exposure, literature reports are scarce, investigating their presence in a variety of tissues and/or diseases, but not specifically addressing estrogen-related diseases or known ZEN effects (e.g. impairment of the metabolism of endo- and xenobiotics). Clearly, additional studies on other potential target organs of this mycotoxin, such as the liver or the spleen, are needed. For example, our target gene prediction analysis suggests an impact on genes related to the immune system, such as interleukin signaling. Indeed, previous studies on splenic and hepatic mRNA expression experimentally confirmed an effect of ZEN on various genes involved in the inflammatory response^54,55^. Yet, when comparing those results to our set of predicted target genes, we could not find any direct overlap. This observation might stem from the tissue-specific expression of microRNAs^15^, which naturally influences the output of target gene prediction. Hence, more research is needed to decipher the role and contribution of microRNAs in ZEN-mediated effects, and subsequently, to conclude on potential similarities and/or discrepancies in the mode of action between ZEN and other (xeno)estrogens.

Since the observation that circulating microRNAs are remarkably stable in body fluids, a lot of attention have been garnered toward establishing their potential use as non-invasive biomarkers in human research. Many of the current disease biomarkers are circulating proteins, but microRNAs offer many advantages over their protein counterparts, such as higher sensitivity, or easier and cheaper for assay development with high specificity^16^. The fact that they can be detected in circulation but released by distant organs make them promising markers for diagnostic and prognostic to monitor the body’s pathophysiological status, such as in estrogen-related diseases^16^. Yet, the number of reports directly comparing the microRNA response to an endocrine disruptor in tissue and serum, thus establishing a specific linkage between these two matrices, is limited. This research gap is even more evident for livestock species, and respective studies for mycotoxins are completely lacking. In pigs, confirmation of suspected cases of ZEN-induced disorders is still challenging, even in the presence of typical signs of hyperestrogenism^12^. On the one hand, feed analysis for ZEN might be inconclusive due to exchange of feed lots, inadequate sample collection or limited analysis spectrum. On the other hand, reliable biomarkers, which allow a sound diagnosis of ZEN-mycotoxicosis in the field, are missing. Direct quantification of ZEN and its metabolites in biological specimens is in principle possible but results greatly vary in dependence on factors such as feed intake, individual differences in metabolism, or sampling time point^13^. In blood, levels of ZEN are often too low for appropriate quantification^56^. Most of all, a correlation of those potential biomarkers of exposure to clinical effects, e.g. increase of uterus weight, could not be established so far^13^. Other biological measures affected in ZEN-exposed pigs, such as hematological or biochemical parameters, lack reproducibility between studies and specificity (summarized by^12^).

To address this issue, we selected 44 microRNAs based on the most relevant results seen after uterus sequencing, and investigated their expression in the serum of piglets. Overall, the effects of ZEN on circulating microRNAs were markedly less pronounced compared to the results in the uterus. Moreover, for several microRNAs the serum and tissue responses were not consistent, showing upregulation in one matrix and downregulation in the other matrix. These observations are in line with a study from Tao *et al*.^57^, in which authors evaluated the effect of weaning stress on the microRNA expression in the jejunum and serum of piglets. Out of 29 microRNAs affected in both serum and jejunum, only five showed a consistent response and similar expressional changes. Reasons for divergent results in serum are nicely reviewed by Voglova *et al*.^58^, and include selective microRNA secretion from the affected tissue, different sources for circulating microRNAs (most predominantly from blood cells and other organs), and lack of disease specificity for some microRNAs.

We first attempted to correlate the results from the uterus and the serum for the 14 microRNAs commonly affected in both ZEN medium and ZEN high groups, but the correlation was rather low (except for ssc-miR-542-3p). As previously mentioned, different sources (e.g. other tissues affected by ZEN) might contribute for the circulating microRNAs, and this could partly explain why better trends were seen for other microRNAs such as ssc-miR-129a, ssc-miR-135a-5p, ssc-miR-432-5p, or ssc-miR-493-3p. Therefore, we assessed next whether a set of microRNAs could be used as non-invasive biomarkers to discriminate between non-exposed and ZEN-exposed piglets, regardless of the ZEN concentration. This was achieved with the use of the ROC curve analysis following the establishment of ratios between the most relevant up- and down-regulated microRNAs. ROC curve analysis revealed several ratios, including ssc-miR-455-5p/ssc-miR-493-3p, ssc-miR-135a-5p/ssc-miR-432-5p, ssc-miR-542-3p/ssc-miR-493-3p capable of discriminating properly between piglets non-exposed and exposed to ZEN. Thus, present data point in the direction that not a single microRNA, but rather a panel of multiple microRNA (ratios) might be used to assess the impact of ZEN in pigs. However, results need to be evaluated in the light of used ZEN concentrations, which were (except for ZEN low) above contamination levels commonly found in the field. Since this work represents a pioneer study in this field, dietary ZEN concentrations were primarily chosen to allow a first assessment on whether ZEN induces a microRNA (dose-) response. Although this approach proved suitable for our purposes, it is subject to the inherent limitation of restricted extrapolation to practically relevant ZEN concentrations. Additionally, we cannot exclude that ZEN elicits a time-dependent effect on serum microRNAs. Although investigated at a metabolic level, it was suggested that ZEN alters serum parameters only in the initial phase of toxin exposure in prepubertal gilts^59^. Thus, the single time-point measurement of serum microRNAs at the end of our experiment represents a limitation of our study, and should be addressed in follow-up works. Consequently, further studies are crucial to confirm our results, and test the microRNA biomarker candidates at lower contamination levels, and different time points.

To conclude, in the present experiment we combined targeted and untargeted transcriptomics approaches to investigate the impact of ZEN on microRNA expression in pigs. Results indicate that ZEN has no influence on the global microRNA response in the jejunum, whereas several uterine microRNAs were significantly and dose-dependently altered in response to ZEN. This effect was partly reflected in serum of exposed animals, thus opening a new field for mycotoxin biomarker discovery. However, further studies both *in vitro* and *in vivo* are essential to clarify the interaction of ZEN and microRNAs, and to fully evaluate the potential of microRNA biomarker candidates.

## Methods

### Animal experiment

The animal experiment was approved by the office of the Lower Austrian Region Government, Group of Agriculture and Forestry, Department of Agricultural Law (approval code LF1-TVG-39/017-2015). The experiment was carried out at the Center for Animal Nutrition (Waxenecker KEG, Austria). All related procedures were performed in compliance with the European Guidelines for the Care and Use of Animals for Research Purposes^60^ and the Austrian Law for Animal Experiments.

Weaned female crossbred piglets (sow: Landrace × Large White, boar: Pietrain; 30 ± 2 days old) were obtained from a local producer. Piglets were housed on slatted floor pens under controlled environmental conditions and had free access to water and feed. After an acclimatization period of seven days, twenty-four piglets were allocated to one of four treatment groups (n = 6). The body weight and vulva area were balanced between groups. Piglets received for 28 days either an uncontaminated basal diet (*Control*) or feed artificially contaminated with 0.17 mg/kg ZEN (*ZEN low*), 1.46 mg/kg ZEN (*ZEN medium*), or 4.58 mg/kg ZEN (*ZEN high*).

Treatment diets were formulated according to age-specific requirements of piglets. For artificial contamination of diets, solid ZEN with a purity of 99.2% was obtained from Fermentek LTD (Israel). Homogenous distribution of ZEN within the diets was achieved by preparation of maltodextrin premixes, which contained appropriate amounts of ZEN and were added to the basal feed at an inclusion rate of 0.9%. Dietary ZEN levels in the groups ZEN low, ZEN medium, and ZEN high as well as the absence of significant ZEN contamination in the Control group (<30 ppb) were confirmed by high performance liquid chromatography – mass spectrometry analysis (Romer Labs GmbH, Austria). Natural contamination of final diets with other major mycotoxins was marginal (deoxynivalenol 166 ppb, fumonisin B_1_ 44 ppb) or absent (aflatoxin B_1_, T-2 toxin, ochratoxin A, ergot alkaloids).

During the experimental period, the general condition and behavior of the piglets were checked daily. In addition, piglets were weighed weekly. The vulva length and width of piglets was measured in regular intervals with a caliper. The vulva area was calculated by multiplying the vulva length with the width. On day 28, blood was collected from individual piglets by punctuation of the *Vena cava cranialis*. After centrifugation (1500 × g, 20 min), serum samples were stored at −80 °C. Following blood sampling, piglets were anesthetized by intramuscular injection of ketamine (0.1 mL/kg b.w. Narketan®, Vétoquinol GmbH, Germany) and azaperone (0.03 mL/kg b.w. Stresnil®, Janssen-Cilag, Germany), and euthanized by intracardial injection of T61® (0.1 mL/kg b.w., Intervet, Germany). The whole reproductive tract was collected and weighed. Small pieces of the corpus uteri and the mid-jejunum were dissected, placed into 1 mL of RNAlater (Ambion Inc., USA), stored overnight at 4 °C, and transferred to −80 °C until transcriptomics analysis.

### microRNA analysis in tissues

#### microRNA extraction and library preparation

Uterus and jejunum samples of 18 animals (six from Control, four each from ZEN low, ZEN medium, and ZEN high; four pigs out of six for the ZEN groups were selected based on the best RNA quality, RIN, obtained after RNA extraction) were used for investigation of microRNA profiles. Approximately 30 mg of tissue were disrupted via a bead-beating step, and the total RNA, including RNA from approximately 18 nucleotides upwards, was extracted and purified with the miRNeasy Mini Kit (Qiagen, Germany). The concentration of isolated RNAs was estimated on a NanoDrop 2000 spectrophotometer (Thermo Scientific, USA), and RNA quality (RIN) with an Agilent 2100 Bioanalyzer (Agilent Technologies, Germany). In total, 1 µg of total RNA was used for construction of small RNA sequencing libraries with the NEBNext Multiplex Small RNA Library preparation set for Illumina (New England Biolabs Inc, USA). Adapter ligated total RNA was reverse transcribed into cDNA, which served as template for PCR amplification (15 cycles) using Illumina’s SR Primer and Index Primers 1–18. Barcoded DNA libraries were purified using the QIAQuick PCR purification protocol (Qiagen, Germany) and quantified by DNA 1000 high sensitivity chip (Agilent Technologies, Germany). Purified samples were pooled at equimolar (50 nM) concentration. Size selection was performed using gel purification to select for insert sizes between 18 and 50 nucleotides. The final multiplexed (n = 18) libraries were sequenced on a NextSeq. 500 sequencing instrument according to the manufacturer´s instructions. Raw data were de-multiplexed and FASTQ files for each sample were generated using the bcl2fastq software (Illumina inc.). FASTQ data were checked using the FastQC tool (http://www.bioinformatics.babraham.ac.uk/projects/fastqc/).

#### RNA-Seq bioinformatic analysis

Total reads were adapter trimmed using Cutadapt and quality filtered (Q-Score > 30) using the FastQC tool. Trimmed and quality filtered reads were aligned to reference microRNA sequences in miRBase release 21 (www.mirbase.org)^24^ using bowtie2. Subsequently, reads were mapped against the *Sus scrofa* genome assembly Sscrofa10.2 as well as Ensembl small non-coding RNA reference databases. Reads were normalized to the total read count per library to generate tags per million (TPM) values. Exploratory and differential expression analyses were performed with microRNAs with a TPM > 10 in the datasets from uterus and jejunum. Clustvis was used for unsupervised clustering analysis. EdgeR^61^ was used for differential expression analysis based on TPM values. The p-values obtained were adjusted for multiple testing using Benjamini-Hochberg false discovery rate (FDR).

#### RNA-Seq validation

For verification of RNA-Seq results, the uterine expression of five selected microRNAs was analyzed by qPCR. Starting from the same RNA samples used for sequencing analysis, cDNA was synthesized via the Universal cDNA Synthesis Kit II (Exiqon, Denmark) using 50 ng of total RNA. PCR amplification was performed in a 96-well plate format in a Roche LC480 II instrument (Roche, Germany) and EXiLENT SYBR Green mastermix (Exiqon, Denmark) with the following settings: 95 °C for 10 min, 45 cycles of 95 °C for 10 s and 60 °C for 60 s, followed by melting curve analysis. To calculate the cycle of quantification values (Cq-values), the second derivative method was used. Cq-values were normalized to U6 snRNA by subtracting the individual microRNA Cq-value from the Cq-value of U6 snRNA for that sample.

#### Target gene prediction

The target gene prediction analysis was done with miRCarta^62^ that integrates information about predicted and experimentally validated microRNA targets from various sources, such as miRTarBase (last update 2017), microT-CDS (last update 2014) and TargetScan (last update 2018). In addition, the results generated from the online database miRDB^63^ (last update 2016) were implemented in our data analysis. A global score was attributed to each target gene found based on two parameters, the prediction score from each source (microT-CDS, TargetScan, and miRDB) and the occurrence score. The prediction score was normalized for each source to reach a score between 0 and 10. The final prediction score was an average of the three scores. The occurrence score was established according to the presence/absence of the predicted target gene in the four sources (10 was the maximum if found in the four sources and 4 the minimum if reported in only one source), and a factor of 2 was included. The global score was ranged between 8 and 30, with 30 being very likely this gene is a target for the microRNA studied. However, due to the large output of gene prediction for some microRNAs, we filtered out the genes below a score of 17. This cut-off was determined as an average of the geometric means of the global score of all predicted genes for each microRNA (ranging from 15.9 to 18.5). We further evaluated the biological functions of the potential target genes by performing GO terms (Gene Ontology Consortium powered by PANTHER) and Reactome pathway annotations when at least one target gene was found in common in the prediction datasets of two microRNAs. Besides, interconnections between microRNA-target genes predictions were generated with the online tool miRNet^64^ (https://www.mirnet.ca/miRNet/faces/home.xhtml) through network-based visual analysis.

### microRNA analysis in serum

Based on the RNA-Seq results of uterus, 44 microRNAs were selected for qPCR analysis in serum of all animals (n = 23, one sample from ZEN low excluded due to hemolysis, OD 414 nm >1). Following centrifugation at 12,000 g for 5 min to remove any cellular debris, 200 μL of serum was mixed with 1000 μL Qiazol and 1 μL synthetic Spike-Ins (Exiqon, Denmark), and RNA was isolated using the miRNeasy Mini Kit (Qiagen, Germany). We synthesized cDNA using the Universal cDNA Synthesis Kit II (Exiqon, Denmark). In total, 2 μL of purified RNA were used per 10 μL RT reaction. The further procedure (RT efficiency, presence of impurities, PCR amplification, calculation and normalization of Cq-values) was carried out as described under 4.2.3., except for using a 384-well plate format with custom Pick&Mix plates (Exiqon, Denmark).

### Statistical and ROC analysis

Statistical and ROC curve analysis, and AUC determination were performed using GraphPad Prism version 7 for Windows (GraphPad Software, La Jolla California USA).

Two-way repeated measures ANOVA followed by Tukey’s multiple comparison test was used for analyzing the data from vulva area measurements. One-way ANOVA followed by Tukey’s multiple comparison test was used for analyzing the data from the reproductive tract weight.

The Pearson correlation coefficient was also determined as a measure of the linear correlation between qPCR fold changes and NGS fold changes.

ROC curves were used to evaluate the discriminatory power of the selected microRNA ratios^65^. The area under a ROC curve quantifies the overall ability of the test to correctly discriminate between non-exposed and exposed individuals to ZEN (poor test when area = 0.50, perfect test when area = 1.00; AUC cannot be below 0.50). The ROC curve evaluation is completed with the report of a P value testing the null hypothesis that the AUC really equals 0.50.

## Supplementary information

Supplementary Information

## Data Availability

All replicates sequencing reads used in this study have been submitted to Gene Expression Omnibus (GEO) under the accession number GSE126989.
