# Supplementary material for: MicroRNAs in porcine uterus and serum are affected by zearalenone and represent a new target for mycotoxin biomarker discovery
Source: Sci Rep. 2019 Jun 28;9:9408. doi: 10.1038/s41598-019-45784-x (PMC6598998; doi:10.1038/s41598-019-45784-x)

Supplementary material

MicroRNAs in porcine uterus and serum are affected by zearalenone and represent a new target for mycotoxin biomarker discovery

Bertrand Grenier [1,*], Matthias Hackl [2], Susanna Skalicky [2], Michaela Thamhesl [1], Wulf-Dieter Moll [1], Roger Berrios [3], Gerd Schatzmayr [1], Veronika Nagl [1]

[1] BIOMIN Research Center, Technopark 1, 3430 Tulln, Austria

[2] TAmiRNA GmbH, Muthgasse 18, 1190 Vienna, Austria

[3] BIOMIN Holding GmbH, Erber Campus 1, 3131 Getzersdorf, Austria

[*] Corresponding author. E-mail address: bertrand.grenier@biomin.net

Supplementary Table S1. Putative or predicted microRNAs significantly affected in uterus of ZEN exposed piglets. MicroRNAs highlighted in bold were significantly affected in the ZEN medium and ZEN high groups (FDR adjusted p-value < 0.05). The log_2(fold change) is indicated for each experimental group with n=4 for ZEN exposed groups and with n=6 for the Control group.

microRNA ID	log_2(fold change) compared to Control			FDR adjusted p-value compared to Control		
	ZEN low	ZEN medium	ZEN high	ZEN low	ZEN medium	ZEN high
put-miR-177	-0.93	1.04	2.10	0.50	0.43	0.046
put-miR-173	-3.69	-1.06	-0.90	0.04	0.43	0.653
put-miR-159	-0.96	-1.65	-2.01	0.59	0.22	0.048
put-miR-152	-1.17	-1.88	-2.10	0.51	0.22	0.048
put-miR-132	-1.37	-1.29	-2.13	0.50	0.38	0.048
hsa-miR-135b-5p	-1.67	-1.80	-2.23	0.34	0.22	0.046
put-miR-136	-1.51	-1.33	-2.62	0.42	0.32	0.001
**put-miR-300**	**-1.34**	**-2.64**	**-2.80**	**0.50**	**0.02**	**0.008**
put-miR-148	-1.48	-1.56	-2.82	0.50	0.32	0.046
put-miR-212	-1.55	-2.06	-2.84	0.48	0.22	0.008

Supplementary Table S2. List of microRNAs analyzed in serum. If primers were designed and used in qPCR based on human homologs, the respective microRNA ID and sequence are provided in brackets. MicroRNA sequences were retrieved from miRBase (http://www.mirbase.org/). Expression levels were defined based on Cq values (low > 30, intermediate 30-33, high < 30).

microRNA ID	Porcine microRNA sequence	Expression in serum
ssc-miR-1	uggaauguaaagaaguaugua	low
ssc-miR-22-3p	aagcugccaguugaagaacugu	intermediate
ssc-miR-22-5p	aguucuucaguggcaagcuuua	intermediate
ssc-miR-34a	uggcagugucuuagcugguugu	low
ssc-miR-125a	ucccugagacccuuuaaccugug	intermediate
ssc-miR-125b	ucccugagacccuaacuuguga	high
ssc-miR-127	ucggauccgucugagcuuggcu	low
ssc-miR-129a	aagcccuuaccccaaaaagcau	low
ssc-miR-133a-5p	agcugguaaaauggaaccaaau	low
ssc-miR-135 (hsa-miR-135a-5p)	uauggcuuuuuauuccuauguga (uauggcuuuuuauuccuauguga)	low
ssc-miR-136	acuccauuuguuuugaugaugga	low
ssc-miR-140-3p	uaccacaggguagaaccacggac	high
ssc-miR-142-3p	uguaguguuuccuacuuuaugg	high
ssc-miR-143-5p	ggugcagugcugcaucucugg	no amplification
ssc-miR-146b	ugagaacugaauuccauaggc	low
ssc-miR-181c	aacauucaaccugucggugagu	low
ssc-miR-182	uuuggcaaugguagaacucacacu	intermediate
ssc-miR-183	uauggcacugguagaauucacug	low

ssc-miR-187 (hsa-miR-187-3p)	ucgugucuuguguugcagccgg (ucgugucuuguguugcagccgg)	no amplification
ssc-miR-195 (hsa-miR-195-5p)	uagcagcacagaaauauuggc (uagcagcacagaaauauuggc)	low
ssc-miR-204	uucccuuugucauccuaugccu	low
ssc-miR-206	uggaauguaaggaagugugugga	low
ssc-miR-335	ucaagagcaauaacgaaaaaug	intermediate
ssc-miR-369	aauaauacaugguugaucuuu	low
ssc-miR-378	acuggacuuggagucagaaggc	high
ssc-miR-424-5p	cagcagcaauucauguuuugaa	high
ssc-miR-432-5p	ucuuggaguaggucauugggu	low
ssc-miR-450a	uuuugcgauguguuccuaauau	low
ssc-miR-450b-5p	uuuugcaauauguuccugaaua	low
ssc-miR-450c-5p	uuuugcgauguguuccuaauac	low
ssc-miR-455-3p	gcaguccaugggcauauacac	low
ssc-miR-455-5p	uaugugccuuuggacuacaucg	intermediate
ssc-miR-486	uccuguacugagcugccccgag	high
ssc-miR-493-3p	ugaaggucuacugugugccagg	low
ssc-miR-493-5p	uuguacaugguaggcuuucauu	no amplification
ssc-miR-497 (hsa-miR-497-5p)	cagcagcacacugugguuugu (cagcagcacacugugguuugu)	intermediate
ssc-miR-503	uagcagcgggaacaguacugcag	low
ssc-miR-542-3p	ugugacagauugauaacugaaa	low
ssc-miR-708-3p	caacuagacugugagcuucuaga	no amplification
ssc-miR-758	uuugugaccugguccacuaac	low
ssc-miR-7135-3p	aucugucugugucucugagcag	no amplification
hsa-miR-135b-5p	uauggcuuuucauuccuaugugа	no amplification
hsa-miR-301a-5p	gcucugacuuuauugcacuacu	no amplification
put-miR-300	uugcaggaacuugugagucuccua	no amplification

Supplementary Figure S1. Profile of read length (a) and total number of reads (b) following small RNA-Seq of uterus samples from piglets exposed to uncontaminated feed (Control, n=6) or feed containing 0.17 mg/kg ZEN (ZEN low, n=4), 1.5 mg/kg ZEN (ZEN medium, n=4) or 4.6 mg/kg ZEN (ZEN high, n=4).

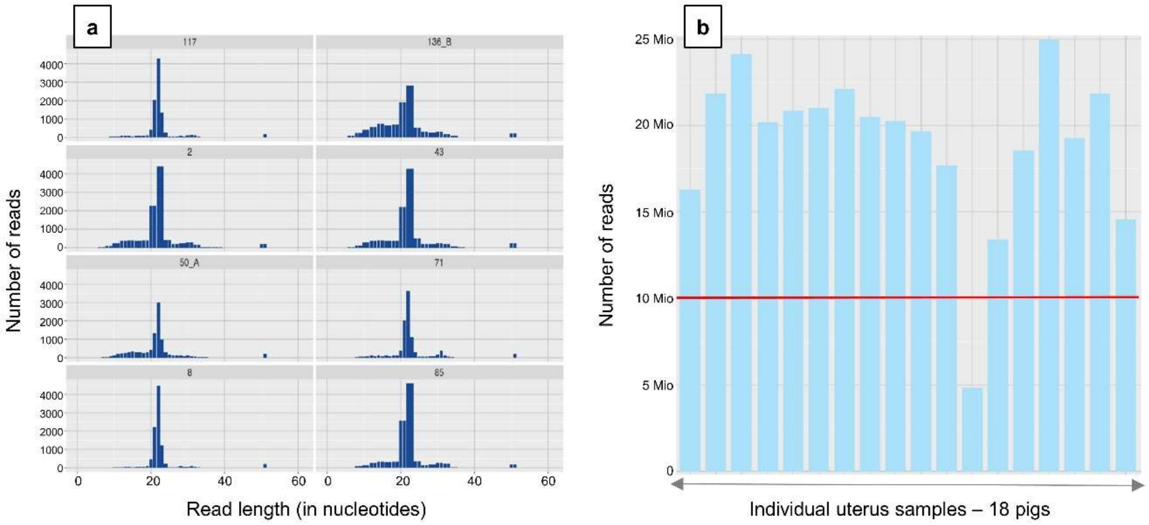

Supplementary Figure S2. Annotation of reads following small RNA-Seq of uterus samples from piglets exposed to uncontaminated feed (Control, n=6) or feed containing 0.17 mg/kg ZEN (ZEN low, n=4), 1.5 mg/kg ZEN (ZEN medium, n=4) or 4.6 mg/kg ZEN (ZEN high, n=4).

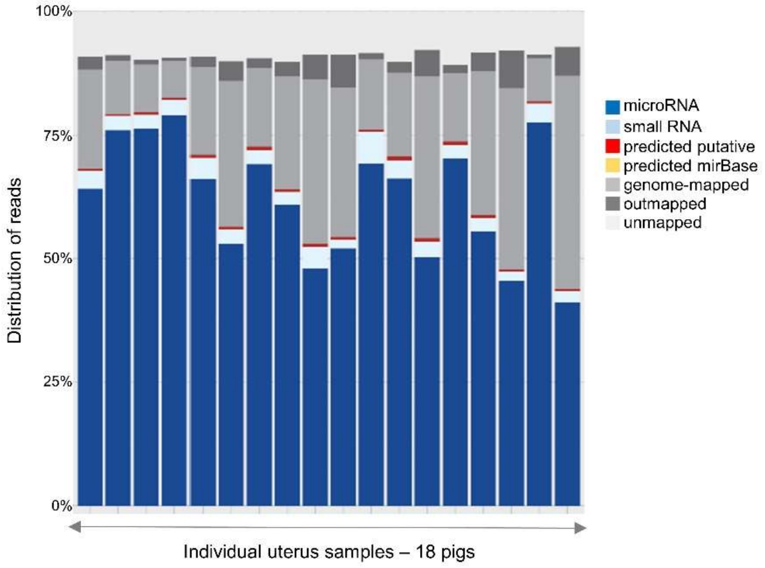

Supplementary Figure S3. Network-based visual analysis of (a) all nodes (912) and edges (1118) connecting the predicted target genes of the microRNAs from the miR-503 cluster (up-regulated in the present study), and of (b) all the nodes (762) and edges (784) connecting the predicted target genes of the microRNAs miR-181c-5p, miR-204-5p, miR-135a-5p (down-regulated in the present study; miR-187-3p was not found part of this network). Networks were generated with the online tool miRNet - https://www.mirnet.ca/miRNet/faces/home.xhtml. Nodes include both target genes and microRNAs, and edges include nodes + number of connections that a node might have with other nodes. Number of edges ≥ number of nodes.

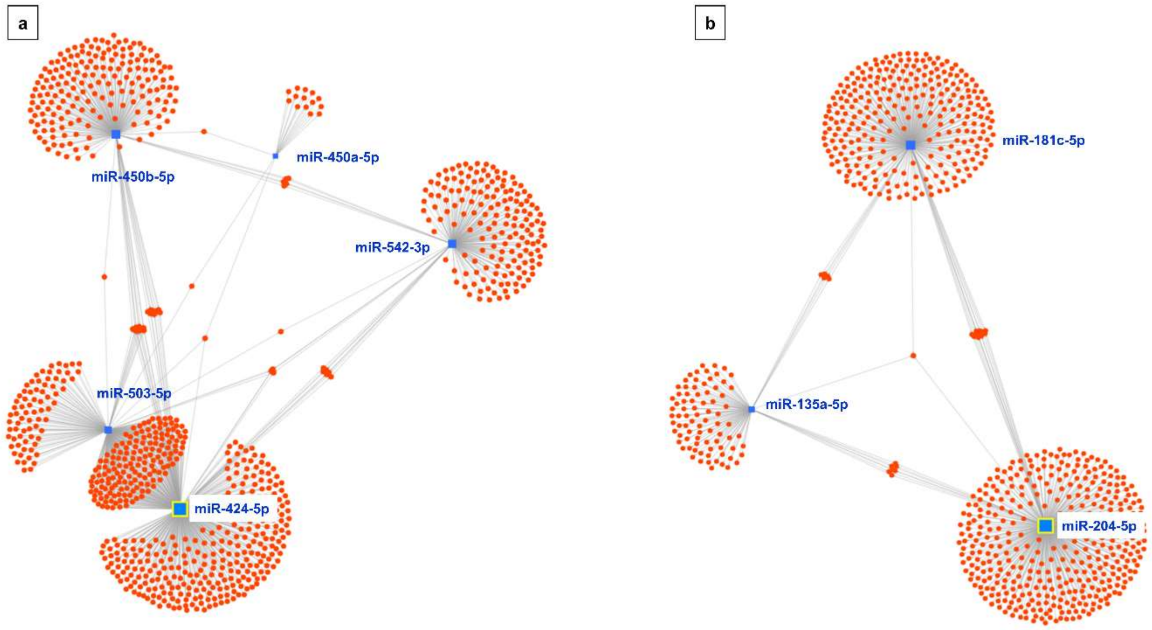

Supplement: Supplementary file 1 — Supplementary Information [file 41598_2019_45784_MOESM1_ESM.pdf]
